# Biosynthesis of long chain base in sphingolipids in animals, plants and fungi

**DOI:** 10.2144/fsoa-2019-0094

**Published:** 2019-11-14

**Authors:** Ryuichi Mashima, Torayuki Okuyama, Mari Ohira

**Affiliations:** 1 Department of Clinical Laboratory Medicine, National Center for Child Health & Development, 2-10-1 Okura, Setagaya-ku, Tokyo 157-8535, Japan

**Keywords:** biosynthesis, degradation, long chain base, sphingolipids

## Abstract

Long chain base (LCB) is a unique building block found in sphingolipids. The initial step of LCB biosynthesis stems from serine:palmitoyl-CoA transferase enzyme, producing 3-ketodihydrosphingosine with multiple regulatory proteins including small subunit SPT a/b and orosomucoid-like protein1-3. 3-Ketodihydrosphingosine reductase and sphingolipid Δ4-desaturase, both of them poorly characterized mammalian enzymes, play key roles for neurological homeostasis based on their pathogenic mutation in humans. Ceramide synthase in mammals has six isoforms with distinct phenotype in each knockout mouse. In plants and fungi, sphingolipids also contain phytosphingosine due to sphingolipid C4-hydroxylase. In contrast to previous notion that dietary intake might be its major route in animals, emerging evidences suggested that phytosphingosine biosynthesis does occur in some tissues such as the skin by mammalian C4-hydroxylase activity of the *DEGS2* gene. This short review summarizes LCB biosynthesis with their associating metabolic pathways in animals, plants and fungi.

## Lay abstract

Sphingolipid is a group of lipids that contains a unique building block known as long chain base (LCB). LCB is susceptible to various biosynthetic reactions such as unsaturation, hydroxylation and methylation. A failure of these enzymatic reactions leads to the pathogenesis in humans with an elevation of LCB-derived specific biomarkers. Herein, we summarized emerging evidences in mammalian LCB biosynthesis in sphingolipids. Some unique metabolic pathways in plants and fungi were also discussed.

Long chain base (LCB) is a derivative of sphingosine, also known as (2S,3R,4E)-2-amino-octadec-4-ene-1,3-diol [1]. LCB is a building block commonly found in all sphingolipids such as ceramide, glycosphingolipids, spingosine-1-phosphate and sphingomyelin. LCBs are generated from multiple enzyme reactions sharing a common biosynthesis pathway in animals [2], plants [3] and fungi [4] and [5]. As illustrated, the biosynthesis pathway involved in LCBs can be classified as *de novo* pathway, recycling pathway and salvage pathway (Figure 1) [6]. The *de novo* pathway in sphingolipids occurs in the endoplasmic reticulum (ER) where ceramide is generated by multiple enzyme reactions initiated with serine:palmitoyl-CoA transferase (SPT). The recycling pathway involves the generation of ceramide from sphingosine and fatty acyl-CoAs catalyzed by ceramide synthase (CerS). The salvage pathway is found in the late endosome/lysosome where degradation of glycosphingolipids occurs at acidic conditions.

**Figure 1.**
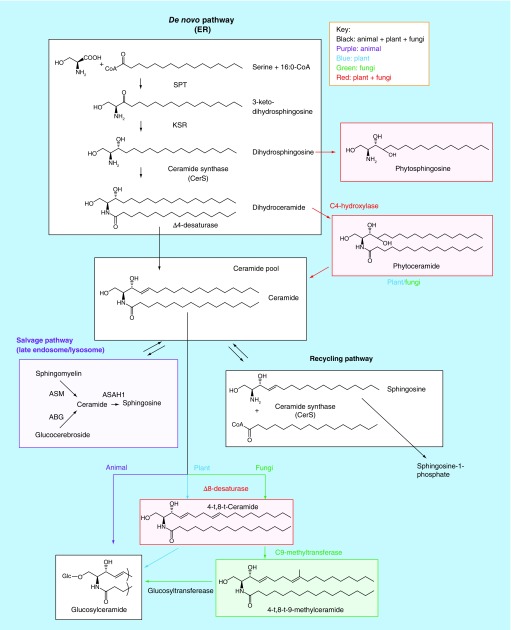
Biosynthesis of long chain bases in sphingolipids. Condensation of serine and C16:0-CoA by SPT followed by reduction by KSR gives rise to dihydrosphingosine via 3-ketodihydrosphingosine as an intermediate compound. Then, *N*-acylation of dihydrosphingosine by CerS followed by Δ4-desaturation gives rise to ceramide in *de novo* pathway. Ceramide is directly glucosylated in animals (black), whereas Δ8-desaturation and C9-methylation might occur prior to glucosylation in plants (blue) and fungi (green), respectively. Phytoceramide might be generated by Δ4-desaturase in plants and fungi (red). Note that ceramide may be generated through *N*-acylation of sphingosine with fatty acid by CerS by recycling pathway and hydrolysis of polar group at 1-position by salvage pathway in the late endosome/lysosome (purple). ABG: Acid β-glucosidase; ASAH1: Acid ceramidase 1; ASM: Acid sphingomyelinase; CerS; Ceramide synthase; KSR: 3-ketodihydrosphingosine reductase; SPT: Serine:palmitoyl-CoA transferase; t: trans.

The physiological role of sphingolipid has been extensively studied in animals, plants and fungi [1,7]. Ceramide is found in all these species in relatively low amounts, playing a key role in signaling function for apoptosis (Table 1). Glycosphingolipids have a variety of functions depending on the structure of oligosaccharides. Among them, glucosylceramide (GlcCer) is a predominant sphingolipid in plants, thus changes in GlcCer levels severely influence their biological property under various physiological conditions such as dried environment, low temperature and strong wind condition. In mammals, however, glycosphingolipids including GlcCer are minor components in sphingolipids, thus one of its biological functions accounts for a receptor for cell–cell or microbial–host interaction, where a high affinity interaction is required. LCBs are also found in other sphingolipids that act as a signaling lipid mediator. One example includes sphingosine-1-phosphate (S1P): this compound has an LCB with a phosphate at the C-1 position [8]. Impaired S1P biosynthesis leads to an attenuation of T-cell mobilization, thus its analog such as FTY720 is used as an immunosuppressant [8]. Apart from these signaling molecules, sphingomyelin is widely found in animals with the highest percentage in sphingolipids. In fact, egg yolk and bovine milk contain high sphingomyelin content compared with other tissues. Because sphingomyelin biosynthesis occurs in the Golgi, which is unique to animals, ceramide in the ER needs to be transferred through a ceramide transfer protein termed CERT [9]. Besides the *de novo* and recycling pathway, the salvage pathway occurs in late endosomes/lysosomes under acidic conditions in conjunction with autophagy. A failure of lysosomal function mostly due to a monogenic mutation in lysosomal hydrolases leads to lysosomal storage disorders with neurovisceral manifestations [6].

**Table 1.** Relative presence of the sphingolipids in animals, plants and fungi.

Sphingolipids	Animals	Plants	Fungi
Ceramide	+	+	+
GlcCer	+	++	++
S1P	+	−	−
Sphingomyelin	++	−	−

+: Presence; −: Absence; GlcCer: Glucosylceramide; S1P: Sphingosine-1-phosphate.

## LCB biosynthesis in animals

Animals have a sophisticated system for the biogeneration of LCBs. Most predominantly found animal LCBs are sphingosine while dihydrosphingosine may be detectable as a minor LCB. Dihydrosphingosine is generated from 3-keto-dihydrosphingosine by enzymatic action of 3-ketosphinganine reductase followed by *N*-acylation with CerS to give rise to dihydroceramide. The formation of ceramide from dihydroceramide is catalyzed by sphingolipid Δ4-desaturase. Occasionally, LCB’s may be detected as a free form such as sphingosine. This compound is the precursor of S1P, which is easily detectable at higher concentration in blood plasma with a potential signaling function *in vivo*.

## SPT

SPT [EC 2.3.1.50] is a rate-limiting enzyme of sphingolipid metabolism locating at the ER [10,11]. SPT catalyzes the condensation of serine and palmitoyl-CoA to produce 3-ketodihydrosphingosine in the presence of pyridoxal 5’-phosphate (vitamin B6) as a cofactor. In mammals, SPT1 and SPT2 has been considered to play a key role for enzyme activity. SPT3 is homologous to SPT2 with a possible complex formation to SPT1 at the ER [10]. In the human genome, SPT2 contains a conserved lysine residue at position 379 that faces cytosol. This amino acid is also found in SPT3, but not in SPT1, thus SPT2 and SPT3 have been considered to have an evolutionally conserved origin. It is widely accepted that mammalian SPT generates C18-LCB predominantly, whereas bacterial SPT exhibits a rather broad specificity for acyl-CoA. For example, relative activity of *Sphingomonas* SPT for myristoyl-CoA and stearoyl-CoA is 75 and 51% of palmitoyl-CoA, respectively [12]. Myriocin is a natural compound that inhibits SPT through a complex formation with pyridoxal 5’-phosphate at the active site of SPT [13,14]. An immunosuppressant FTY720 was developed based on myriocin.

Enzyme activity of SPT is regulated by multiple mechanisms. Tsc3p is an 80 amino acid yeast protein that positively regulates SPT enzyme activity [15]. Absence of Tsc3p leads to a temperature-sensitive phenotype and this is rescued by supplementation of 3-ketosphinganine as well as related compounds. Subsequently, yeast two-hybrid analysis using a human brain library identified small subunit SPT a (ssSPTa) and its homolog ssSPTb as hLCB1-LCB2a-interacting proteins [16]. Importantly, ssSPTa/b enhances SPT activity similar to Tsc3p, while no homology was recognized between ssSPTa/b and Tsc3p. Biochemical studies revealed that ssSPT reacts with C16-CoA exclusively, while ssSPTb reacts with C18-CoA at suboptimal level. The *Steller* mutant mice were identified by abnormal shiny flecks in the fundus induced by nitrosourea-induced mutagenesis at the Jackson Laboratory [17]. Genomic sequence analysis revealed that these mice have a H56L mutation in ssSPTb, a conserved amino acid found in human ssSPTb, mouse ssSPTa and fish ssSPTb, respectively. These mice exhibited an enhanced C20-LCB production, neurodegenerative phenotype and aberrant membrane structure in retina. Neural phenotype seems to be highly associated with enhanced expression of ssSPTb, because knockout mice harboring a *lacZ* gene under the control of ssSPTb promoter showed its neural localization.

Orosomucoid-like proteins (ORMDL) are negative regulators for SPT activity [18]. These proteins were originally found by bioinformatic analysis [19]. There are three genes such as ORMDL1-3 in humans and its two orthologs Orm1/2 in yeast. Expression analysis revealed that these proteins are ER proteins. Subsequent study identified that yeast Orm1 and Orm2 attenuates SPT activity using a yeast mutant orm 1Δorm2Δ strain [20]. When the accumulating phytosphingosine was compared with that in WT yeast, the former exhibited a 4.8-fold higher activity. Similar results were found in mammalian cells using a combination of siRNA for ORMDL1-3, establishing that they act as negative regulators for SPT activity [21,22].

Given that SPT plays an essential role in maintaining either the survival signaling or cell membrane integrity during entire development in animals, the failure of this would lead to the deleterious outcomes. This assumption is supported by the fact that the null mutant of either the *Sptlc1* or *Sptlc2* gene dies in embryo in mice (Table 2) [23]. Intriguingly, the heterozygote mutant for these genes such as *Sptlc1*(+/-) or *Sptlc2*(+/-) showed a reduced level of plasma ceramide and liver SPT activity, whereas these animals have normal triglycerides, cholesterol and sphingomyelins in plasma, suggesting that SPT might be involved in the control of atherosclerotic development. In *Caenorhabditis elegans*, the null mutant showed the extended life span compared with wild-type [24]. In this model, SPT could be linked to a suppressor of apoptosis, because the expression of genes involved in programmed cell death was enhanced.

**Table 2.** Phenotype of null or pathogenic mutants of long chain base-synthesizing enzymes.

Enzyme	Animal			Plant	Fungi	
	Human	Mice	*Caenorhabditis elegans*	*Arabidopsis thaliana*	*Saccharomyces cerevisiae*	*Candida albicans*
SPT [EC. 2.3.1.50]	HSAN1 (OMIM: #162400) [25,26]	Lethal in *SPTLC1*(-/-) or *SPTLC2*(-/-) mice [23]; ↓SPT activity, ↓Ceramide in *SPTLC1*(+/-) or *SPTLC2*(+/-) mice [23]; Peripheral neuropathy in SPTLC1 p.C133W mutant mice [27]; Neural degeneration and ↑C20-LCB in Stellar mice [17]	Extended life span [24]	Lethal [28,29]; ↓male gametophyte development [28]; ↓Size, abnormal morphology [29]	Lethal [30]	Not reported
KSR [EC 1.1.1.102]	Progressive symmetric erythrokeratoderma (OMIM: #136440) [31]	Not reported	Not reported	Not reported	Not grow normally [32]	Not reported
Δ4-desaturase [EC. 1.14.19.17]	Hypomyelinating leukodystrophy (OMIM: #615843) [33]	↓Life span in null mice [34]; ↓Dex-Induced insulin resistance in *Des1*(+/−) mice [34]	Extended life span [35]	↓GlcCer [36]	Not reported	Not reported
C4-hydroxylase [EC. 1.14.18.5]	Not reported	Not reported	Not reported	↑Sphingolipids [37]; ↓Size, cell expansion, division in double knockout [37]; ↑Dwarfing [37], ↓Transition from vegetative to reproductive growth [37]	↓t18:0 [38]	Not reported
Δ8-desaturase (sphingolipid 8-[E]-desaturase, EC 1.14.19.18 and sphingolipid 8-[E/Z]-desaturase, EC 1.14.19.29)	Not reported	Not reported	Not reported	↓GlcCer, ↑GIPC, ↓Cold stress [39]	Not reported	↓Morphological change [40]
C9-methyltransferase [EC. 2.1.1.317]	Not reported	Not reported	Not reported	Not reported	Not reported	Resistance to plant defensin [41]; ↓Hypal elongation [42]

↑: Increase; ↓: Decrease; Dex: Dexamethasone; GlcCer: Glucosylceramide; HSAN1: Hereditary sensory and autonomic neuropathy, type 1; KSR: 3-ketodihydrosphingosine reductase; LCB: Long chain base; SPT: Serine:palmitoyl-CoA transferase.

Emerging evidence has suggested SPT might be associated with neurological disease progression. Hereditary sensory and autonomic neuropathy, type 1 (HSAN1; Online Mendelian Inheritance in Man [OMIM]: #162400) is a dominantly inherited sensorimotor axonal neuropathy with onset in the first or second decades of life [43]. In 2001, two groups identified that the gene responsible for this disorder is the *SPTLC1* gene [25,26]. Interestingly, all mutations found for these patients are missense mutations, but not frameshifts and nonsense mutations. Based on these genetic data, a biochemical study revealed that a SPT1 p.C133W mutation led to an accumulation of 1-deoxy-sphingosine and 1-deoxymethy-sphinganine by SPT with palmitoyl-CoA and glycine and alanine, rather than serine, respectively [44]. Consistently, overexpression of the wild-type SPT1 improved HSAN1-like phenotype observed in a transgenic mice expressing SPT1 protein with C133W mutation, suggesting that these LCBs are pathogenic with an uncharacterized mechanism [45]. There is a biochemical paper investigating the currently reported mutations for HSAN1 [46]. Although the development of HSAN1 has been thought to be associated with an impaired SPT activity, this paper reported that some pathogenic mutations such as SPT1 p.S331F/Y and SPT2 p.I505Y showed an enhanced SPT activity with an abnormal enzyme reaction product such as C20-sphingosine.

## 3-Ketodihydrosphingosine reductase

Ketodihydrosphingosine reductase (KSR) [EC 1.1.1.102] catalyzes the reduction of 3-ketodihydrosphingosine that gives rise to dihydrosphingosine. Progressive symmetric erythrokeratoderma is a Mendelian disorder of cornification that severely affects skin (OMIM: #617526). A recent report identified five affected individuals with compound heterozygosity for KSR mutation in humans [31]. Affected individuals showed an expansion of filaggrin immunostaining, suggesting a defect in keratinocyte terminal differentiation. This manifestation can be improved by retinoic acid treatment, raising a possibility that either recycling pathway or salvage pathway, which do not require KSR-dependent ceramide production, might be activated (Figure 1). In zebrafish, KSR mutation leads to steatosis and hepatic injury due to S1P accumulation with concomitant elevation of sphingosine and sphinganine [47].

## Sphingolipid Δ4-desaturase

Sphingolipid Δ4-desaturase [EC 1.14.19.17] involves the reaction of a *trans* double bond formation between C4 and C5 of dihydrosphingosine [48,49]. Importantly, this animal enzyme does catalyze desaturation of dihydroceramide, but does not of dihydrosphingosine. In other words, sphingosine is produced only from ceramide through *N*-deacylation by CerS in recycling pathway in the Golgi, but not from dihydrosphingosine in *de novo* synthesis pathway in the ER. The reported phenotype of *Degs1*(-/-) mice in 2007 involves an increased ratio of dihydroceramide to ceramide, reduced life span, scaly skin, sparse hair and tremor [34]. Although *Degs1*(+/-) mice grow comparably with wild-type mice, these mice have an impaired insulin resistance induced by glucocorticoid, saturated fat and obesity. More recently, a report studying individuals with hypomyelinating leukodystrophy in humans identified that the *DEGS1* gene is involved in this disorder (36). The reported mutations in humans relate to either missense, frameshift or nonsense, all of them found in exons 2 and 3. Alternatively, no large deletions such as recombination and intronic frameshift were reported. More than half of the cases related to consanguine marriage. Interestingly, some lysosomal storage disorders such as Krabbe disease and metachromatic leukodystrophy also show similar MRI phenotype to this *DEGS1*-mediated hypomyelinating leukodystrophy, thus the salvage pathway could be involved in this pathogenesis. The phenotype in humans was ensured by the fact that DEGS1 morpholino-injected zebrafish *Danio rerio* showed a decreasing myelin basic protein with an impaired movement as found in humans.

Human sphingolipid C4-hydroxylase, namely hDES2, has been initially identified in keratinocyte [50]. This enzyme catalyzes hydroxylation of [^3^H]*N*-octanolydihydrosphingosine, demonstrating clearly that dihydroceramide can be a substrate. The expression of mRNA was most abundant in the skin with a lesser amount in the brain, kidney, large and small intestine, respectively. It is largely accepted that phytosphingosine detectable in mammals may be derived from dietary origin; however, some evidence suggests that phytosphingosine might be biosynthesized by an enzymatic action of hDES2 [51]. Due to the fact that phytosphingosine increases the expression of several genes for sphingolipid biosynthesis, such as SPT, CerS3, ELOVL4, thus biosynthesis of phytoceramide seems to be tightly regulated [52]. There is a study reporting that DEGS2 polymorphism is linked to schizophrenia [53], raising a possibility that pathogenic alteration of sphingolipid metabolism could be linked to the neurological phenotype that was also observed in DEGS1 [33] and SPT and its regulatory proteins as found in HSAN1 and ssSPTb-mutated stellar mice [17,44].

## CerS

Mammalian CerS [EC 2.3.1.24] has six isoforms with distinct substrate specificity with *N*-acyl chain length (41). A biochemical study revealed that C14-C18 ceramide are formed by CerS1, CerS5 and CerS6 [54]. CerS2 seems to be a unique enzyme with a higher activity for very long chain fatty acids such as C22-C26. Apart from these five isoforms, CerS3 *N*-acylates various lengths of fatty acids including ultra-long chain fatty acids (≥C26) which is often found in skin ceramides [55]. A knockdown experiment of CerS1-6 showed that CerS2 is involved in C22-C24 *N*-acylation, while others are involved in C14-C18 *N*-acylation in MCF-7 cells [54]. To gain insight into the molecular mechanism, a mutagenesis study reported that the substrate specificity of CerS is associated with 11 residues in the putative last two transmembrane domains of CerS [56].

The phenotype of all CerS-deficient mice has been reported (Table 3). Overall, these may be attributed to either an abnormal regulation of cell death that leads to neurodegeneration and carcinogenesis, S1P-mediated immunoregulation or uncharacterized mechanism. Among six isoforms, CerS2 and CerS3 have distinct physiological roles. Mice lacking CerS2 show skewed accumulation of C16-ceramide with normal ceramide content in total in the liver, demonstrating that other CerS, such as CerS5, acts cooperatively [57]. An elevated S1P level in CerS2-deficient mice is associated with an accumulation of its precursor sphingosine, thus CerS2 plays a key role to maintain sphingosine at low levels by *N*-acylating sphingosine with C22-26 fatty acids, the best substrates for CerS2, but not for other isoforms. CerS3 is often found in the skin and testis: this enzyme has a broad specificity for fatty acyl-CoA including ≥C26 species. Mice lacking CerS3 show an impaired skin formation with dried body due to an increasing trans-epidermal water loss, a manifestation with a pathologically enhanced evaporation of water from the skin, leading to neonatal death after birth [55]. In fact, wet body weight of CerS3-deficient mice was significantly lower than that of wild-type mice, while both show comparable dry body weight. Finally, some phenotypes such as cerebellar atrophy, reduced life span and an increased ratio of dihydroceramide to ceramide reported in CerS-deficient mice were also observed in *Degs1*-deficient mice (Tables 2 & 3). Thus, CerS enzymes involve both *de novo* and recycling pathways; the former plays a key role in the pathogenesis in these disorders.

**Table 3.** Phenotype of ceramide synthase-deficient mice.

CerS isoform	Phenotype	Ref.
CerS1	Neurodegeneration↑	[58]
CerS2	C22-24 ceramide↓	[57,59]
	Myelin sheath↓, cerebellar degeneration↑, hepatocarcinoma↑	[60]
	Reduced life span	[61]
	Pheochromocytoma	[62]
	S1P-dependent thymocyte egress↑	[63]
	LPS-induced septic shock↑	[64]
	EAE exacerbation↓	[65]
	Renal sulfatide↓, phytosphingosine containing sulfatide↓, urinary pH→	[66]
CerS3	Trans-epidermal water loss↑, die within 3–4 h after birth	[55]
CerS4	Hair loss↑	[67]
	Unaltered S1P-dependent thymocyte egress	[63]
CerS5	Diet-induced obesity↓	[68]
CerS6	Inflammation in DSS↑	[69]
	EAE exacerbation↑	[70]
	Diet-induced obesity↓	[71]
	Behavioral abnormality↑	[72]

↓: Decrease;↑: Increase; →: Unaltered; CerS: Ceramide synthase; DSS: Sodium dextran sulfate; S1P: Sphingosine-1-phosphate.

## Sphingosine kinase

Sphingosine kinase [EC 2.7.1.91] catalyzes the conversion of sphingosine into S1P, regulating the cellular survival, proliferation, differentiation, migration and immune function [73]. There are two isozymes such as sphingosine kinase 1 and 2. Sphingosine kinase 1 is a cytosolic protein also found near the plasma membrane, whereas sphingosine kinase 2 predominantly localizes at intracellular membrane, such as mitochondria, ER and the nucleus [74]. S1P binds to five isoforms of S1P receptors (S1PR1-5), all of them are G protein-coupled receptors with different components. Among them, S1PR1-3 shows a broad expression. S1PR4 is mainly detectable in leukocytes, whereas S1PR5 is found in oligodendrocytes in the brain. S1P-mediated activation of S1PR1 on mature lymphocytes mediates their egress from the thymus, indicating that S1P-mediated biological action might be primarily linked to the amount of expressed receptors on the target cells [75].

## S1P lyase

S1P lyase (SPL) [EC. 4.1.2.27] is the final enzyme in the sphingolipid degradative pathway [76]. It converts S1P to *trans*-2-hexaecenal and ethanolamine phosphate, respectively. Deficiency of SPL leads to hyperlipidemia because very-low-density lipoprotein, low-density lipoprotein and high-density lipoprotein in plasma were elevated in mice [77]. This reason seems to be associated with an enhanced expression of the Pparg gene, a master regulator of lipid biosynthesis. Accumulating data for whole-exon sequencing revealed that SPL is involved in nephrosis, ichthyosis, adrenal insufficiency or neurologic defects and immunodeficiency, collectively called as SPL insufficiency syndrome (SPLIS) [76]. A hallmark alteration of biomarkers for SPLIS involves an elevation of sphingolipids in the blood and fibroblasts of affected individuals.

## Unique LCB biosynthesis pathways in plants & fungi

Apart from animals, plants and fungi produce phytosphingosine, a trihydroxylated LCB (Figure 1). These species have additional enzymes that introduces a double bond in LCB, which is also absent in animals.

## Sphingolipid C4-hydroxylase

In yeast, C4-hydroxylase enzyme [EC 1.14.18.5] is encoded by *SUR2* gene, which was originally identified through a functional screening [78]. Yeast SUR2 protein has a similar sequence of cytochrome *b*_5_ that contains an oxo-diiron catalytic center. Roles of SUR2 protein in yeast include an increase in resistance of cells to the *Pseudomonas syringae* cyclic lipodepsipeptide syngomyocin, an inhibitor for ergosterol synthesis pathway [78]. Separately, the deletion of *SUR2* gene in yeast suppresses the Ca^2+^-sensitivity phenotype. The rationale behind these might be, at least in part, attributed to the C4-OH-mediated membrane fluidity [79].

Gene expression analysis revealed that there are two genes for sphingolipid C4-hydroxylase enzymes in *Arabidopsis thaliana*, such as the *SBH1* and *SBH2* [37]. These are found globally with an enhanced expression of SBH1 over SBH2 in stem, leaf, root, seedling, silique and flower. Consistently, *sbh1* null mutant exhibited an enhanced accumulation of total LCB content compared with *sbh2* null mutant [37]. A complete loss of C4-hydroxylase activity in double mutants and a partial loss in *sbh1* null mutant resulted in activity-dependent reduction of size due to defective cell expansion and division. Under these conditions, the genes involved in programmed-cell death were highly expressed, suggesting that ceramide with dihydrosphingosine and sphingosine may induce apoptotic signals. The gene responsible for C4-hydroxylase is often found in multiple copies in the genome. For example, *A. thaliana* and *Oryza sativa* have two and five distinct C4-hydroxylase enzymes, possibly due to genome duplication often found in plants [37,80].

## Sphingolipid Δ8-desaturase

Chen *et al.* reported in 2012 that sphingosine Δ8-desaturase activity is encoded by two genes such as the *SLD1* and *SLD2* in *Arabidopsis* [39]. While a mutant-lacking SLD1 protein showed a more severe phenotype compared with those lacking SLD2 protein, there appeared to be only a marginal alteration of phenotype even in double mutant. Apparent phenotypes included 50% reduction of GlcCer and altered susceptibility to lower temperature stress. Although the precise reason for rather mild phenotype of this double mutant remains uncertain, one explanation could be associated with the enhanced expression of both SLD1 and SLD2 proteins in flower, whereas other tissues such as root, leaf and stem, respectively, have a rather limited expression of this enzyme in *Arabidopsis* [39]. In fact, a mutant of Δ8-desaturase of tomato *Solanum lycopersicum* showed an impaired resistance on chilling conditions [81]. This suggests that the development of a gain-of-function mutant for sphingolipid Δ8-desaturase might be an intriguing target for food science research. Genome editing technology is an emerging technique that allows us to manipulate the genome of interest, leading to add valued biological feature for humans to mutants.

A recent bioinformatic study profiling 20 sphingolipid Δ8-desaturase genes in 12 plants with an additional two yeast genes suggested that these sphingolipid Δ8-desaturase enzymes might be classified into three distinctive species in plants and fungi [82]. Biochemically, sphingolipid Δ8-desaturase in yeast generate 8-*trans*-LCBs, while plant enzymes usually produce a mixture of 8-*cis*- and 8-*trans*-LCBs with a distinct ratio [82,83]. Based on the sequences of fungi enzymes such as KlD8 (*Kluyveromyces lactis*, AB085690.1) and PpaD8 (*Pichia pastoris*, AY700777), phylogenetic analysis of plant enzymes was performed. As the result, one group of genes that gives rise to 8-*trans*-isomer at higher reaction rate could be linked to yeast origin, while another group that gives rise to 8-*cis*-isomer at lower reaction rate would be associated with higher plant-derived ancestor [83]. Although it is unknown why plant Δ8-desaturase that selectively generate 8-*cis*-LCB exhibits genetically high similarity to yeast enzymes those produce 8-*trans*-LCB. A possible explanation could be that the immediate genetic modification involving substrate specificity might occur when fungi Δ8-desaturase gene transmitted to ancestor plant. Today, sphingolipid Δ8(E)-desaturate (EC 1.14.19.18) and sphingolipid Δ8(E/Z)-desaturate (EC 1.14.19.29) are found as sperate entities in EC classification.

## LCB biosynthesis pathway specific to fungi

Similar to animals and plants, fungi LCBs are generated through SPT with palmitoyl-CoA and serine as the substrates [84]. The best characterized example includes yeast SPT which requires Tsc3p as the regulatory factor. SPT is also found in *Bacteroides* and *Sphingomonas*. Fungi SPT exhibits a rather broad substrate specificity for free fatty acids [12].

## C9-Methyltransferase

Prominent feature of fungi LCB is the presence of C9-methylated LCBs, an enzymatic reaction product of C9-methyltransferase [EC 2.1.1.317] [4]. In order to identify this gene, the genome sequence of several fungi strains, such as *Candida albicans, Cryptococcus neoformans, Magnaporthe grisea* and *Neurospora crassa* that express this enzyme, were compared with those which lacks this enzyme [85]. Once sequence data were obtained, C9-methyltransferase gene was further identified in other fungi strains such as *Debaryomyces hansenii, Kluyveromyces lactis, Saccharomyces kluyveri* and *Yarrowia lipolytica* [42]. C9-methyltransferase activity seems to be required for some limited fungi species. For example, a mutant lacking this enzyme in *Fusariumgraminearum* showed a severe growth defect with an impaired C9-methylated GlcCer concentration [85,86]. In separate study, it was shown that this enzyme is only necessary for the filamentous fungi such as *C. albicans*, but dispensable for vegetative growth of yeast [42].

## Future perspective

Accumulating evidence has indicated that SPT activity is highly regulated by multiple factors. First, the activity of SPT is regulated by ssSPTa/b and ORMDL1-3 in human: the former proteins activate SPT while the latter attenuate SPT activity. Second, although mammalian SPT generates C18-LCB from C16-CoA and serine, ssSPTb increases C20-LCB formation from C18-CoA in the presence of wild-type SPT. Third, both glycine and alanine are capable to react with SPT particularly when it is mutated, leading to the generation of 1-deoxy- and 1-deoxymethyl-LCB. Because SPT localizes in the ER membrane, which hampers detailed mechanistic study, we need to better understand the molecular interaction of SPT and other associating regulatory proteins in native state in the future.

A recent discovery that the *DEGS1* gene is associated with hypomyelinating leukodystrophy in humans provides an insight into how sphingolipids are acting in animals. Interestingly, patients defective in the *DEGS1* gene showed cerebellar atrophy, reduced life span and an increase in ratio of dihydroceramide to ceramide, all these phenotypes are reported in Degs-1 and CerS-deficient mice (Tables 2 and 3). Some lysosomal storage disorders such as Krabbe disease and metachromatic leukodystrophy also show similar MRI phenotype to this *DEGS1*-mediated hypomyelinating leukodystrophy, thus the further understanding of an initial event leading to pathogenesis needs to be explored.

The bioactivity of lipids including LCBs, at least in part, depends on the geometry (i.e., *trans* and *cis*) and position of double bond. Lipidomics technology allows us to profile a variety of lipids, providing information for LCB biosynthesis. A successful application reported a wide LCB distribution in fungi [87,88]. Based on this technique, future studies will be able to detect a variety of sphingolipid species more precisely and easily. For example, the current collision-induced ionization technique generates relatively large fragmentation ions, which might be suitable for detailed structural information. An emerging ionization technique such as an electron impact method could be applicable to LC–MS, allowing the generation of a more detailed fragmentation pattern [89]. An intriguing application of lipidomics for clinical application includes the detection of microbiota-derived sphingolipids in host specimen. For example, *Bacteroides* is anaerobic bacteria found in human gut that generates sphingolipids. Thus, if we could have a sophisticated methodology for its quantification, these microbiota-derived biomarkers might provide any clinically useful information for immune response, malignancy and neural response induced by gastrointestinal stimuli in humans.

In conclusion, although the principle of LCB biosynthesis appears to be well understood, many emerging evidence will improve our understanding of LCB biology. As can be found in HSAN1 and SPLIS, when a growing amount of clinical sequencing data becomes available, yet to emerge treatment options for these rare disorders might be developed.

**Executive summary**

- Serine:palmitoyl-CoA transferase (SPT) is a rate-limiting enzyme of sphingolipid biosynthesis, that is regulated by small subunit SPT a/b and orosomucoid-like protein1-3 in humans. While both small subunit SPT a/b increases long chain base (LCB) production by SPT, ssSPTb makes SPT produce C20-LCB more abundantly. SPT also accepts glycine and alanine as an alternative of serine, the best characterized substrate. This is particularly true when it is mutated, leading to an accumulation of pathogenic sphingolipids such as 1-deoxyl- and 1-deoxymethyl-LCBs.
- Recent advances in whole-genome sequence analysis of clinical samples demonstrated that SPT and sphingosine-1-phosphate lyase are closely associated with rare genetic disorders such as hereditary sensory and autonomic neuropathy, type 1 and SPL insufficiency syndrome. Based on the accumulating clinical sequencing data, such a trend might continue to develop more rapidly in the future.
- Plants and fungi have a unique LCB biosynthesis system. Although mammalian LCBs have sphingosine as the most abundant LCBs, plants have phytosphigosine and/or sphingosine with an additional double bond between C8 and C9. Furthermore, some, but not all, fungi have odd-numbered LCBs when C9-methyltransferase is present.
